# Digital Marker for Early Screening of Mild Cognitive Impairment Through Hand and Eye Movement Analysis in Virtual Reality Using Machine Learning: First Validation Study

**DOI:** 10.2196/48093

**Published:** 2023-10-20

**Authors:** Se Young Kim, Jinseok Park, Hojin Choi, Martin Loeser, Hokyoung Ryu, Kyoungwon Seo

**Affiliations:** 1 Department of Applied Artificial Intelligence Seoul National University of Science and Technology Seoul Republic of Korea; 2 Department of Neurology College of Medicine Hanyang University Seoul Republic of Korea; 3 Department of Computer Science, Electrical Engineering and Mechatronics ZHAW Zurich University of Applied Sciences Winterthur Switzerland; 4 Graduate School of Technology and Innovation Management Hanyang University Seoul Republic of Korea

**Keywords:** Alzheimer disease, biomarkers, dementia, digital markers, eye movement, hand movement, machine learning, mild cognitive impairment, screening, virtual reality

## Abstract

**Background:**

With the global rise in Alzheimer disease (AD), early screening for mild cognitive impairment (MCI), which is a preclinical stage of AD, is of paramount importance. Although biomarkers such as cerebrospinal fluid amyloid level and magnetic resonance imaging have been studied, they have limitations, such as high cost and invasiveness. Digital markers to assess cognitive impairment by analyzing behavioral data collected from digital devices in daily life can be a new alternative. In this context, we developed a “virtual kiosk test” for early screening of MCI by analyzing behavioral data collected when using a kiosk in a virtual environment.

**Objective:**

We aimed to investigate key behavioral features collected from a virtual kiosk test that could distinguish patients with MCI from healthy controls with high statistical significance. Also, we focused on developing a machine learning model capable of early screening of MCI based on these behavioral features.

**Methods:**

A total of 51 participants comprising 20 healthy controls and 31 patients with MCI were recruited by 2 neurologists from a university hospital. The participants performed a virtual kiosk test—developed by our group—where we recorded various behavioral data such as hand and eye movements. Based on these time series data, we computed the following 4 behavioral features: hand movement speed, proportion of fixation duration, time to completion, and the number of errors. To compare these behavioral features between healthy controls and patients with MCI, independent-samples 2-tailed *t* tests were used. Additionally, we used these behavioral features to train and validate a machine learning model for early screening of patients with MCI from healthy controls.

**Results:**

In the virtual kiosk test, all 4 behavioral features showed statistically significant differences between patients with MCI and healthy controls. Compared with healthy controls, patients with MCI had slower hand movement speed (*t*_49_=3.45; *P*=.004), lower proportion of fixation duration (*t*_49_=2.69; *P*=.04), longer time to completion (*t*_49_=–3.44; *P*=.004), and a greater number of errors (*t*_49_=–3.77; *P*=.001). All 4 features were then used to train a support vector machine to distinguish between healthy controls and patients with MCI. Our machine learning model achieved 93.3% accuracy, 100% sensitivity, 83.3% specificity, 90% precision, and 94.7% *F*_1_-score.

**Conclusions:**

Our research preliminarily suggests that analyzing hand and eye movements in the virtual kiosk test holds potential as a digital marker for early screening of MCI. In contrast to conventional biomarkers, this digital marker in virtual reality is advantageous as it can collect ecologically valid data at an affordable cost and in a short period (5-15 minutes), making it a suitable means for early screening of MCI. We call for further studies to confirm the reliability and validity of this approach.

## Introduction

### Background

As the prevalence of Alzheimer disease (AD) is rapidly increasing worldwide [1], it is important to screen early for mild cognitive impairment (MCI), a preclinical stage of AD [2,3]. Whereas AD is an irreversible disease, patients with MCI still have the chance to restore their cognitive function to normal [4,5]. To this end, various biomarkers related to the physiological, pathological, or anatomical characteristics of AD have been studied [6,7]. Cerebrospinal fluid amyloid levels [8-10] and magnetic resonance imaging (MRI) results [11-13], which include representative AD biomarkers, have been used to objectively quantify the early clinical symptoms of patients with AD [14]. However, continuously monitoring these biomarkers is unfeasible as obtaining these data incurs either high cost (eg, MRI) or great inconvenience for the patients due to the invasive nature of the procedure [15-17]. Digital markers, however, that evaluate cognitive impairment by analyzing behavioral data collected from digital devices such as smartphones [18-20] or personal computers [21-23] could alleviate the above challenges and would provide an inexpensive, easy-to-use alternative. To this end, the development and validation of digital markers that allow for the reliable screening of MCI is an important research topic [17,24,25].

Instrumental activities of daily living (IADL) tasks, which represent cognitively complex activities performed in everyday life, are suitable tasks to be developed as digital markers [26,27]. Because successfully carrying out IADL tasks requires a high level of cognitive function, patients with MCI showed significantly poorer IADL performance compared with healthy controls [28,29]. Among various IADL tasks, ordering menu items from a kiosk was found to be the assignment where patients with MCI showed the lowest performance [28,30]. The reason why kiosk-related IADL tasks are significantly impaired in patients with MCI is that the kiosk use scenario requires comprehensive and complex cognitive functions, including memory to recall multiple menu items, attention to focus on the correct menu item among the various menu items, and executive function to carry out an order consecutively [31,32]. This is why, in this study, an IADL task of ordering menu items from a kiosk was used for early screening of patients with MCI.

Virtual reality (VR) technology allows behavioral data to be collected while performing IADL tasks in a controlled environment [33-36]. In addition, as VR provides a fully immersive experience of the real world in a noninvasive way, participants can naturally interact with their virtual environment, allowing ecologically valid real time data to be collected [25,37,38]. Previous studies have shown that analysis of hand movements [39,40], eye movements [41-43], and performance data [44-48] that are collected while performing IADL tasks in VR can be used for early screening of patients with MCI. Seo et al [39] found that patients with MCI showed slower hand movement speed than healthy controls when performing an IADL task of withdrawing money from a bank in VR. Oyama et al [47] differentiated patients with MCI from healthy controls by analyzing eye movement features (in particular, proportion of fixation duration) collected during video and image viewing tasks. Eraslan Boz et al [44] demonstrated that patients with MCI showed significantly poorer performance than a healthy control group when carrying out a virtual supermarket task. This manifested, among other things, in longer completion times and a larger number of errors. Although these studies showed the possibility of early screening of MCI using behavioral data collected during the IADL task in VR, it remains an open question how these multimodal behavioral data can be combined into digital markers. Therefore, Piau et al [15] called for research that could advance digital marker technology by integrating heterogeneous data (ie, hand movements, eye movements, and performance data).

### Objectives

This study aims to develop a “virtual kiosk test” that can evaluate IADL ability and determine its validity as a VR digital marker for early screening of MCI. The virtual kiosk test involves performing a task of ordering menu items from a kiosk in a virtual environment, while behavioral data such as hand movements, eye movements, and performance data are collected in real time. The validity of the virtual kiosk test is determined by feeding these behavioral data to a machine learning model for early screening of MCI, thereby proposing relevant results. The use of the VR digital marker is expected to complement conventional AD biomarkers by collecting ecologically valid data repeatedly at low cost and noninvasively for patients with MCI.

Overall, the objectives of this study can be summarized into 3 main aspects. First, we explored key features that can significantly differentiate patients with MCI from healthy controls among behavioral data collected during the virtual kiosk test (ie, hand movements, eye movements, and performance data). Second, we analyzed the correlation between the conventional neuropsychological test results measuring various domains of cognitive function and the virtual kiosk test results. Third, we developed a machine learning model for early screening of MCI using virtual kiosk test results and validated its early screening performance in terms of accuracy, sensitivity, specificity, precision, *F*_1_-score, and area under the receiver operating characteristic curve (AUC).

## Methods

### Participants

A total of 51 participants were recruited from Hanyang University Hospital, Republic of Korea, using nonprobability consecutive sampling from January to November 2022. Among them, 20 were healthy controls and 31 were patients diagnosed with MCI. Healthy controls were recruited among volunteers at the medical center who met the inclusion criteria. Patients with MCI were randomly recruited among generic outpatients who exhibited relatively declined cognitive function during an annual wellness visit at the Department of Neurology, Hanyang University Hospital. Patients with MCI were diagnosed by 2 neurologists with 18 and 22 years of experience, respectively, according to criteria reported by Albert et al [49]. The inclusion criteria required participants to be over 50 years of age to minimize potential age-related confounding factors in cognitive impairment [50]. Participants had to demonstrate the ability to perceive auditory and visual stimuli and meaningfully interact with a virtual environment. The exclusion criteria for the participants were as follows: (1) inability to read text; (2) medical history of neurodegenerative or psychiatric diseases; and (3) history of any kind of dementia or brain surgery.

### Neuropsychological Tests

A total of 5 neuropsychological tests were administered from the Seoul Neuropsychological Screening Battery-Core (SNSB-C) [51], which has been validated in the Korean population. The SNSB-C was used to assess the following five domains of cognitive function: (1) Digit Span Test: Forward+Backward (DST: F+B) for assessing attention, (2) Short Form of the Korean-Boston Naming Test (S-K-BNT) for language function, (3) Rey Complex Figure Test (RCFT) for visuospatial function, (4) Seoul Verbal Learning Test-Elderly’s Version: Delayed Recall (SVLT-E: DR) for memory, and (5) Korean-Trail Making Test-Elderly’s Version: Part B (K-TMT-E: B) for frontal-executive function.

### Virtual Kiosk Test

**Figure 1:**
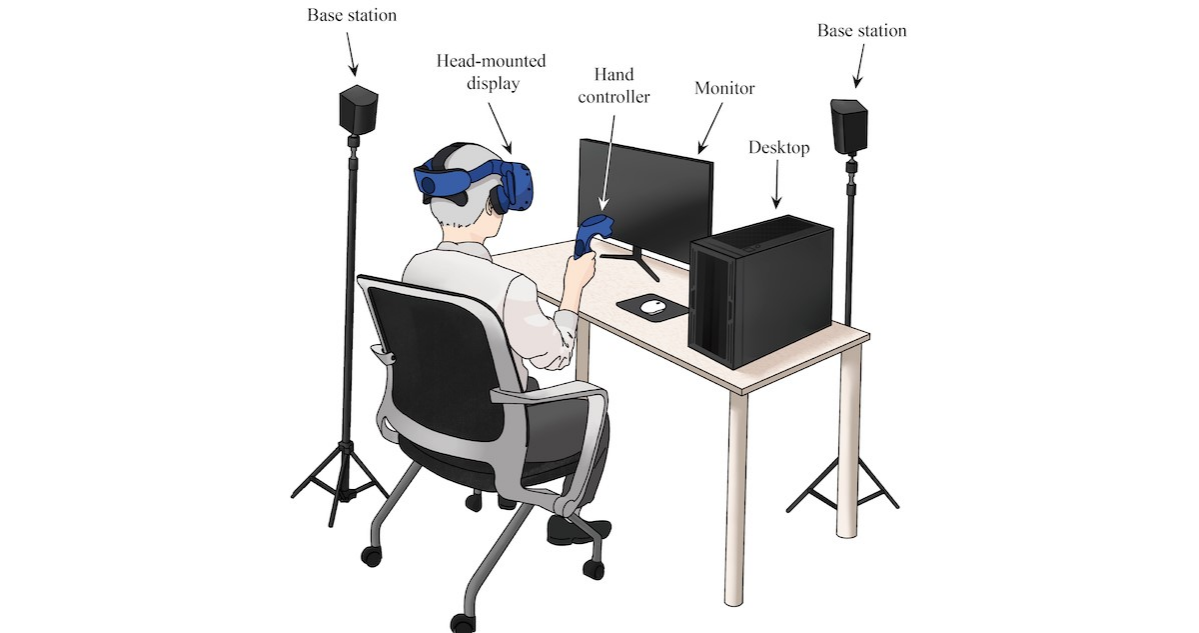
Experimental setup for the virtual kiosk test.

**Figure 2:**
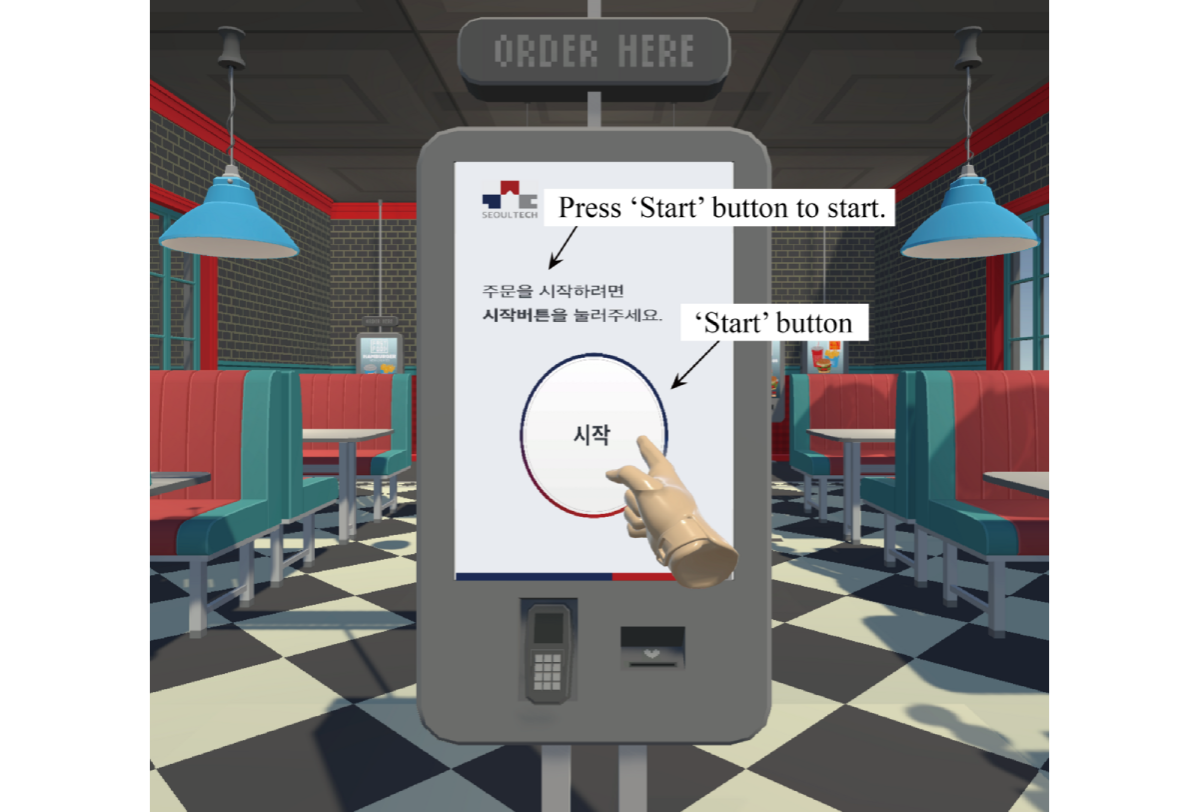
The kiosk and virtual hand in the virtual environment.

The virtual kiosk test records participants’ behavioral data (ie, hand movements, eye movements, and performance data) when performing the IADL task of ordering a menu item using a kiosk in a virtual environment. Figure 1 shows the experimental setup for the virtual kiosk test installed in a 1.3 m×1.3 m×2 m space. Participants sat on a chair for safety, wore a head-mounted display (Vive Pro Eye; HTC), and conducted a virtual kiosk test with a hand controller held in their right hand. A total of 2 base stations were used to measure participants’ hand movements. Figure 2 shows the kiosk and virtual hand in the virtual environment that participants would see when wearing a head-mounted display. Participants’ eye movements were tracked by sensors in their head-mounted display. The virtual kiosk test consists of six sequential action steps: (1) choose a place to eat, (2) choose a burger item, (3) choose a side item, (4) choose a drink item, (5) choose a payment method, and (6) enter a 4-digit payment password (Multimedia Appendix 1 showcases a demonstration video). Participants received the following instructions verbally before performing the virtual kiosk test: “The place to eat is a restaurant. Please order a shrimp burger, cheese sticks, and a Coca-Cola using the kiosk. Please use a credit card as the payment method. The card payment password is 6289.”

During the virtual kiosk test, hand movement data, eye movement data, and performance data were collected. Based on these time series data, the following four behavioral features were derived: (1) hand movement speed, (2) proportion of fixation duration, (3) time to completion, and (4) number of errors. Table 1 shows these 4 features collected by the virtual kiosk test and their corresponding descriptions.

**Table 1:** The 4 behavioral features collected by the virtual kiosk test and their descriptions.

Feature			Description
**Hand movement feature**			
	Hand movement speed (m/s)	The average speed of hand movements collected while a participant performed the virtual kiosk test	
**Eye movement feature**			
	Proportion of fixation duration (%)	The percentage of time a participant focused on the instructed menu item out of all menu items	
**Performance feature**			
	Time to completion (seconds)	The total time a participant took to complete the virtual kiosk test	
	The number of errors	The total number of errors collected while a participant performed the virtual kiosk test	

### Procedures

All participants performed neuropsychological tests and a virtual kiosk test under the supervision of 2 neurologists, one with 18 years and one with 22 years of experience. The order of neuropsychological tests and a virtual kiosk test was counterbalanced. Both tests differed significantly in duration. Whereas a neuropsychological test took about 40 minutes, the virtual kiosk test took only about 5 minutes. Before the virtual kiosk test, participants underwent an eye calibration process and 2 practice sessions to familiarize themselves with the virtual environment interaction. Each practice session was considered complete when participants could perform the entire virtual kiosk test procedure from start to end. These practice sessions typically lasted 2-3 minutes and occasionally extended up to a maximum of 5 minutes. In total, the eye calibration and practice sessions took around 10 minutes. Although participants were given the opportunity to interrupt the virtual kiosk test in case of cybersickness, all of them completed the entire test without any break. Overall, the entire experiment took about 55 minutes.

### Analysis

To evaluate the validity of the virtual kiosk test as a digital marker for early screening of MCI, several statistical analyses were performed using SPSS Statistics (version 27; IBM Corp). First, a chi-squared test and independent-sample 2-tailed *t* tests were conducted to compare the basic demographic characteristics and neuropsychological test results between healthy controls and patients with MCI. Second, independent-sample 2-tailed *t* tests were again performed to compare the virtual kiosk test results between healthy controls and patients with MCI. Third, a multiple regression analysis was performed to explore the relationship between neuropsychological test results and virtual kiosk test outcomes. Bonferroni correction was applied to adjust for multiple comparisons in all statistical analyses. Following these statistical analyses, a machine learning model was trained using significant features collected from the virtual kiosk test to develop a digital marker that can be used to distinguish patients with MCI from healthy controls. To minimize the risk of overfitting, the machine learning process used a stratified train-test split with a 7:3 ratio, where 36 participants (14 healthy controls and 22 patients with MCI) were allocated to the training subcohort and the remaining 15 participants (6 healthy controls and 9 patients with MCI) were placed in the test subcohort. The Python 3 programming language and the “Scikit-learn” library were used for model training and implementation. The machine learning model’s validity was evaluated on the test subcohort, assessing accuracy, sensitivity, specificity, *F*_1_-score, and AUC as performance metrics.

### Ethics Approval

Written informed consent was obtained from participants after a full explanation of the study’s objectives, procedures, potential risks, and benefits. The experimental design and recruitment criteria were approved by the institutional review board of Hanyang University Hospital, Republic of Korea (HYUH-2021-08-020-004).

## Results

### Basic Demographic Characteristics and Neuropsychological Test Results

As shown in Table 2, the basic demographic characteristics (ie, gender, age, and educational level) of healthy controls and patients with MCI were found to have no significant differences, as indicated by both a chi-square test and independent-sample 2-tailed *t* tests. However, independent-sample 2-tailed *t* tests indicated that all results of the neuropsychological tests showed significant differences between healthy controls and patients with MCI.

**Table 2:** Comparison of basic demographic characteristics and neuropsychological test results between healthy controls and patients with mild cognitive impairment.

Characteristic			Healthy controls (n=20)		Patients with MCI^a^ (n=31)		*P* value^b^
**Basic demographic characteristics**							
	Gender (female), n (%)	10 (50)		16 (52)		>.99	
	Age (years), mean (SD)	70.95 (6.02)		72.68 (7.75)		>.99	
	Educational level (years), mean (SD)	12.60 (4.54)		9.32 (4.98)		.16	
**Neuropsychological tests, mean (SD)**							
	DST: F+B^c^ (number of correct answers)	10.60 (1.54)		8.52 (1.82)		<.001	
	S-K-BNT^d^ (number of correct answers)	13.05 (1.43)		11.0 (1.88)		<.001	
	RCFT^e^ (score)	33.28 (2.44)		26.42 (6.56)		<.001	
	SVLT-E: DR^f^ (number of correct answers)	7.15 (2.30)		2.52 (2.59)		<.001	
	K-TMT-E: B^g^ (time to completion)	38.75 (22.93)		114.65 (103.85)		.02	

^a^MCI: mild cognitive impairment.

^b^Adjusted *P* values after the Bonferroni correction (.05/8=.006).

^c^DST: F+B: Digit Span Test: Forward+Backward.

^d^S-K-BNT: Short form of the Korean-Boston Naming Test.

^e^RCFT: Rey Complex Figure Test.

^f^SVLT-E: DR: Seoul Verbal Learning Test-Elderly’s version: Delayed Recall.

^g^K-TMT-E: B: Korean-Train Making Test-Elderly’s version: Part B.

### Differences in Virtual Kiosk Test Results Between Healthy Controls and Patients With MCI

Differences in virtual kiosk test results between healthy controls and patients with MCI were assessed by independent-sample 2-tailed *t* tests (Table 3). A total of 4 features collected from the virtual kiosk test (ie, hand movement speed, proportion of fixation duration, time to completion, and the number of errors) showed significant differences between healthy controls and patients with MCI. In particular, compared with healthy controls, patients with MCI showed significantly slower hand movement speed (*t*_49_=3.45; *P*=.004), a lower proportion of fixation duration (*t*_49_=2.69; *P*=.04), a longer time to completion (*t*_49_=–3.44; *P*=.004), and a greater number of errors (*t*_49_=–3.77; *P*=.001) while performing the virtual kiosk test.

**Table 3:** Comparison of virtual kiosk test results between healthy controls and patients with mild cognitive impairment.

Virtual kiosk test feature		Healthy controls (n=20)		Patients with MCI^a^ (n=31)	*P* value^b^
**Hand movement feature, mean (SD)**					
	Hand movement speed (m/s)		0.23 (0.06)	0.17 (0.05)	.004
**Eye movement feature, mean (SD)**					
	Proportion of fixation duration (%)		56.0 (13.1)	43.7 (17.5)	.04
**Performance feature, mean (SD)**					
	Time to completion (seconds)		40.41 (19.35)	104.95 (82.19)	.004
	Number of errors		1.60 (1.60)	4.16 (2.75)	.001

^a^MCI: mild cognitive impairment.

^b^Adjusted *P* values after the Bonferroni correction (.05/4=.012).

### Correlation Between Neuropsychological Test Results and Virtual Kiosk Test Results

To explore the relationship between the 4 significant virtual kiosk test features (ie, hand movement speed, proportion of fixation duration, time to completion, and the number of errors) and neuropsychological test results, a correlation analysis was conducted using multiple regression (Table 4). Hand movement speed showed significant correlations with attention (DST: F+B), language function (S-K-BNT), visuospatial function (RCFT), and frontal-executive function (K-TMT-E: B). Proportion of fixation duration showed significant correlations with attention (DST: F+B), visuospatial function (RCFT), memory (SVLT-E: DR), and frontal-executive function (K-TMT-E: B). Time to completion showed significant correlations with attention (DST: F+B), visuospatial function (RCFT), and frontal-executive function (K-TMT-E: B). The number of errors showed significant correlations with all 5 domains of cognitive function.

**Table 4:** Correlation analysis between neuropsychological test results and virtual kiosk test results.

Virtual kiosk test feature			DST: F+B^a^		S-K-BNT^b^		RCFT^c^	SVLT-E: DR^d^		K-TMT-E: B^e^
**Hand movement speed**										
	Correlation coefficient	0.36		0.46		0.43		0.28	–0.42	
	*P* value^f^	.047		.003		.007		.22	.01	
**Proportion of fixation duration**										
	Correlation coefficient	0.53		0.30		0.51		0.43	–0.41	
	*P* value^f^	<.001		.16		<.001		.008	.01	
**Time to completion**										
	Correlation coefficient	–0.40		–0.34		–0.49		–0.27	0.46	
	*P* value^f^	.02		.07		.001		.24	.003	
**The number of errors**										
	Correlation coefficient	–0.45		–0.53		–0.62		–0.41	.57	
	*P* value^f^	.004		<.001		<.001		.01	<.001	

^a^DST: F+B: Digit Span Test: Forward+Backward.

^b^S-K-BNT: Short form of the Korean-Boston Naming Test.

^c^RCFT: Rey Complex Figure Test.

^d^SVLT-E: DR: Seoul Verbal Learning Test-Elderly’s version: Delayed Recall.

^e^K-TMT-E: B: Korean-Train Making Test-Elderly’s version: Part B.

^f^Adjusted *P* values after the Bonferroni correction (.05/9=.006).

### Machine Learning Classification Performance Using Virtual Kiosk Test Results

For early screening of MCI, a machine learning model was trained using all 4 significant features extracted from the virtual kiosk test (ie, hand movement speed, proportion of fixation duration, time to completion, and the number of errors), normalized within the range 0 to 1, as this combination yielded the highest accuracy. After experimenting with different models, a support vector machine (SVM) was chosen for this study because it showed the best performance (Table S1 in Multimedia Appendix 2). Model performance was evaluated in terms of accuracy, sensitivity, specificity, precision, *F*_1_-score, and AUC. Optimal SVM hyperparameters (radial basis function kernel, C=1.0; γ=1.86) were found by a grid search. Leave-one-out cross-validation was used to minimize the risk of overfitting.

**Figure 3:**
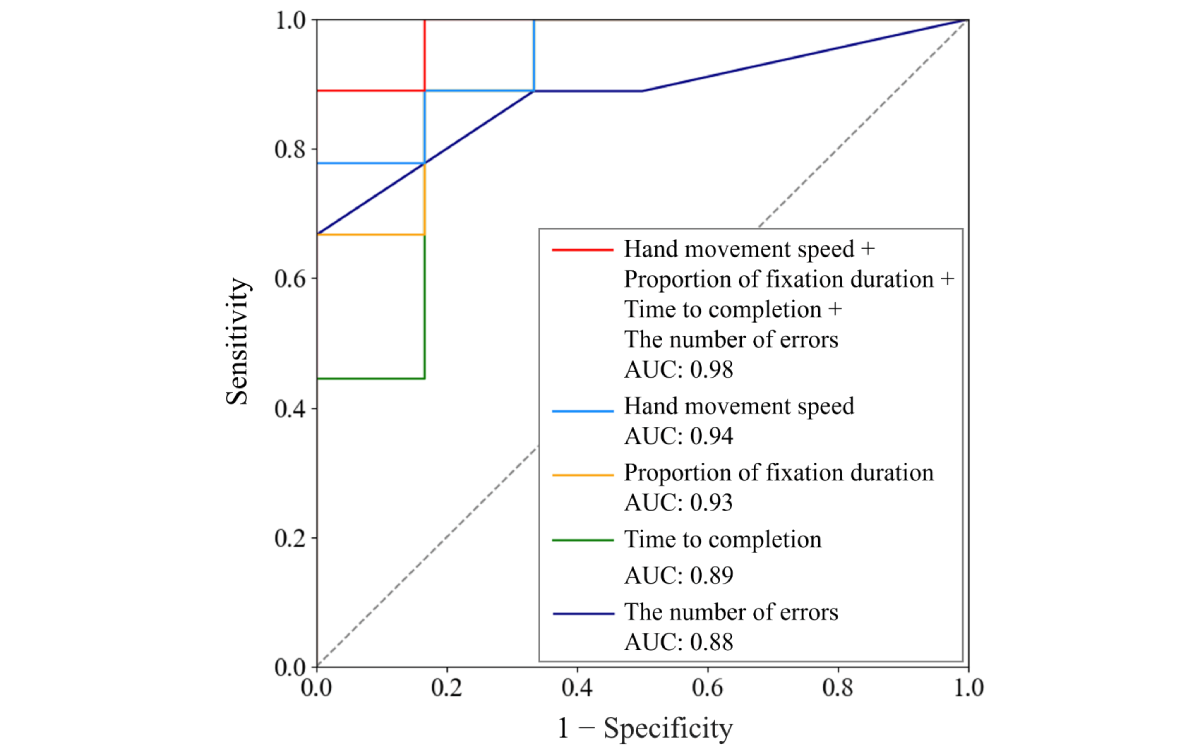
Comparison of receiver operating characteristic curves and the AUC. The best classification performance was obtained when the support vector machine was trained using all 4 features of the virtual kiosk test (ie, hand movement speed, proportion of fixation duration, time to completion, and the number of errors). AUC: area under the receiver operating characteristic curve.

As shown in Figure 3, patients with MCI could best be told from healthy controls when all 4 significant features of the virtual kiosk test (ie, hand movement speed, proportion of fixation duration, time to completion, and the number of errors) were used (93.3% accuracy, 100% sensitivity, 83.3% specificity, 90% precision, 94.7% *F*_1_-score, and AUC=0.98). Training the SVM on only a single feature yielded significantly lower performance, regardless of the feature chosen. Using the hand movement speed as the only feature yielded 80% accuracy, 88.9% sensitivity, 66.7% specificity, 80% precision, 84.2% *F*_1_-score, and AUC=0.94. Replacing that feature by proportion of fixation duration returned 60% accuracy, 100% sensitivity, 0% specificity, 60% precision, 75% *F*_1_-score, and AUC=0.93. Using time to completion as the single feature returned 80% accuracy, 77.8% sensitivity, 83.3% specificity, 87.5% precision, 82.4% *F*_1_-score, and AUC=0.89. Finally, using the number of errors as the only feature yielded 73.3% accuracy, 88.9% sensitivity, 50% specificity, 72.7% precision, 80% *F*_1_-score, and AUC=0.88.

## Discussion

### Principal Findings

The findings of our study suggest that the virtual kiosk test can serve as a valid digital marker for early screening of patients with MCI by measuring IADL-related behavioral data in a virtual environment. Through the virtual kiosk test, we observed that patients with MCI—as compared with a healthy control group—showed significantly slower hand movement speed, lower proportion of fixation duration, longer time to completion, and a greater number of errors while interacting with a kiosk in VR. The best SVM trained on these multimodal behavioral features (ie, hand movement speed, proportion of fixation duration, time to completion, and the number of errors) achieved the highest performance with 93.3% accuracy, 100% sensitivity, 83.3% specificity, 90% precision, 94.7% *F*_1_-score, and 0.98 AUC in early screening of patients with MCI. Additionally, our results demonstrated a strong correlation between these behavioral features and neuropsychological test results, indicating that the virtual kiosk test is an ecologically valid digital marker for evaluating comprehensive cognitive functions in real-world contexts.

**Figure 4:**
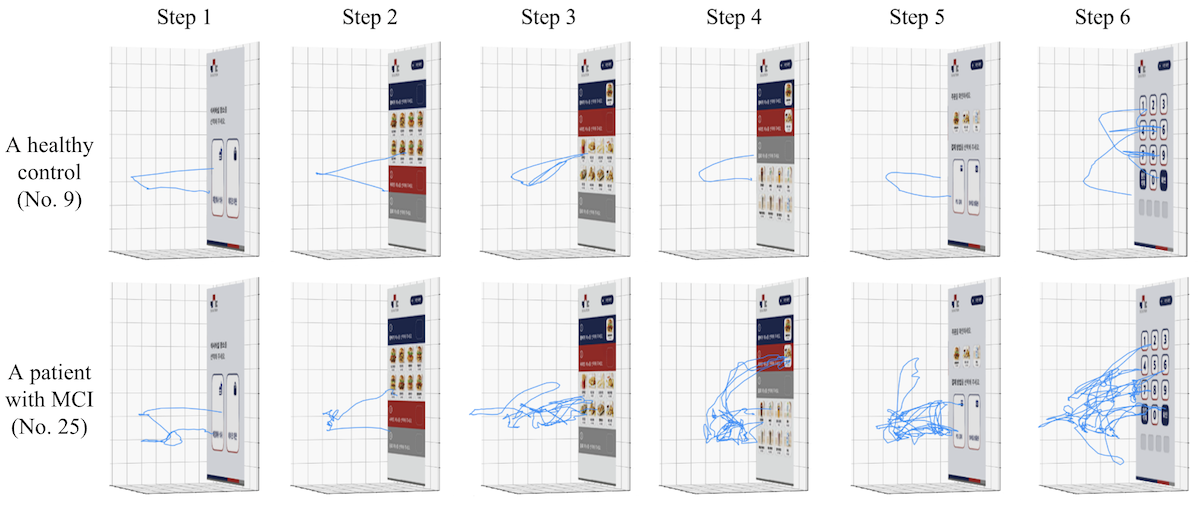
Comparison of hand movements between a healthy control (participant No. 9) and a patient with MCI (participant No. 25) for different virtual kiosk screens. MCI: mild cognitive impairment.

Our findings suggest that analyzing hand and eye movements in VR can be useful for the early screening of patients with MCI. Figure 4 shows that patients with MCI featured distinctive hand movement patterns as compared with healthy controls. Specifically, as shown in steps 3 to 6 in Figure 4, hand movements of patients with MCI became more complex when the kiosk screen displayed a large number of items to choose from. This can be attributed to the strong correlation between hand movements and various cognitive functions, including attention, language function, visuospatial function, and frontal-executive function. For instance, patients with MCI with impaired attention might struggle to focus on target menu items, leading to wandering hand and eye movements around the areas of interest and hesitant actions. Patients with MCI with impaired language function might read menu items slowly, resulting in slower and more complex hand movements. Patients with MCI with visuospatial impairment might find it challenging to plan and coordinate their hand movements, resulting in more complex hand movement patterns. This could explain the hand movements around the wrong menu items that we observed in some cases. Finally, frontal-executive function impairment might hinder patients with MCI from figuring out what to do, resulting in slower and longer hand movements. Overall, measuring and processing hand movement data during the virtual kiosk test improves MCI early screening performance, as hand movement patterns tend to be degraded by cognitive impairment.

**Figure 5:**
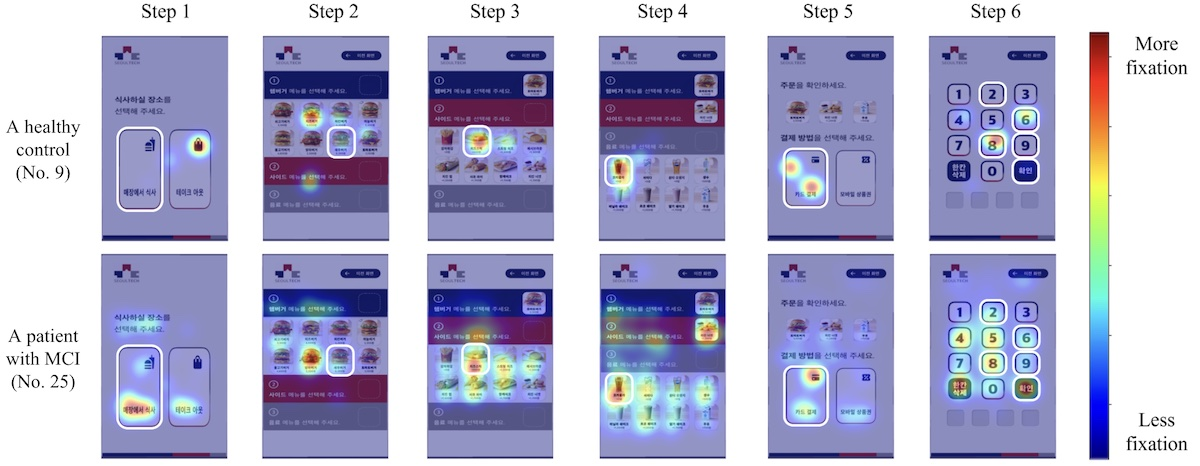
Comparison of eye movements between a healthy control (participant No. 9) and a patient with MCI (participant No. 25). Participants’ areas with more eye fixation are shown in red, and areas with less eye fixation are shown in blue. Target menu items for each step feature a white frame. MCI: mild cognitive impairment.

Figure 5 illustrates that, patients with MCI exhibit markedly different eye movement patterns compared with healthy controls. Patients with MCI frequently become distracted by nontarget menu items on a kiosk screen. Such distracted eye movement patterns in patients with MCI can be explained by impairments in attention, visuospatial function, memory, and frontal-executive function. For example, impaired attention could explain why patients with MCI are easily distracted by nontarget menu items. Figure 5 illustrates these scattered fixation patterns that can be observed at all steps of the test, regardless of the specific screen the patients with MCI currently see. Impaired visuospatial function may make it difficult for patients with MCI to identify the spatial structure of menu items, leading to fixations on a broader range of screen regions and wandering toward nontarget menu items. Impaired memory and frontal-executive function may cause patients with MCI to have trouble recalling target menu items and skimming menu items repeatedly. Overall, eye movements in the virtual kiosk test contribute to improving MCI early screening performance by examining distinctive eye movement patterns that emerge during information processing on a kiosk screen.

The effectiveness of hand and eye movement analysis in VR for early screening of MCI can be explained by the concept of embodied cognition [52-54]. Embodied cognition emphasizes that cognitive function is closely intertwined with action capacities and that assessing the relationship between cognition and action can provide additional insight into detecting early signs of neurodegenerative diseases. By analyzing hand and eye movements during the performance of a cognitively complex IADL task, such as ordering menu items using a kiosk, we could gain information about how cognition and action are tightly coupled. The complex hand movements and distracted eye movements exhibited by patients with MCI in this study can be viewed as digital markers that reflect the characteristics of embodied cognition.

The use of VR digital markers from the virtual kiosk test as a screening tool for MCI offers practical advantages over conventional biomarkers and other digital markers. Previous studies have shown that both conventional biomarkers and VR digital biomarkers demonstrate similar levels of performance, with a pooled sensitivity and specificity of around 70% and 80%, respectively, for hippocampal magnetic resonance imaging and blood biomarkers [55,56] and a pooled sensitivity of 89% and a specificity of 91% for VR digital biomarkers [57]. Despite this similarity, VR biomarkers present distinct benefits, including cost-effectiveness and noninvasiveness, allowing for data collection over an extended period with minimal effort. The virtual kiosk test can be completed in just 5 minutes, making it significantly quicker than the administration of neuropsychological tests, which typically take over 40 minutes. Additionally, our study focused on a single IADL task, yet still achieved high classification performance, likely due to the comprehensive cognitive assessment provided by the kiosk usage scenario. Moreover, our study found that participants enjoyed performing the virtual kiosk test and expressed interest in repeating it, suggesting its potential as a digital therapeutic tool for cognitive rehabilitation in patients with MCI [58-60]. However, further research with a larger sample size is needed to validate this assumption. In conclusion, VR digital markers from the virtual kiosk test are promising candidates that could complement conventional biomarkers and other digital biomarkers in early MCI screening.

### Limitations

This study has several limitations that require further investigation. First, the small nonprobability sample used in this study may introduce bias into the machine learning model, thus limiting its generalizability. The total sample size of 51 participants, with an imbalanced distribution of 20 healthy controls and 31 patients with MCI, was smaller compared with other studies using VR digital markers [36,61]. To address these limitations, future research should use larger sample sizes, gather data through multi-institutional collaborations, and use diverse sampling methods that account for the prevalence of MCI caused by various diseases, such as diabetes [62] and hypertension [63]. Second, although a correlation between virtual kiosk test results and neuropsychological test results was established, additional research is needed to understand the physiological, pathological, or anatomical characteristics of digital markers. Future clinical studies should explore the relationship between the virtual kiosk test and conventional biomarkers, such as MRI. Third, while this study used behavioral features for early screening of patients with MCI based on previous literature, future studies could explore completely new features through deep learning models. More advanced deep learning models, such as convolutional neural networks or recurrent neural networks, could analyze the hand and eye movement patterns of patients with MCI from a spatial or temporal perspective. Lastly, while the statistical validity of the virtual kiosk test was demonstrated in this study, further data collection is necessary before it can be used in clinical practice. Collecting clinical standard data will establish clear thresholds for early screening of MCI using the virtual kiosk test in clinical settings.

### Conclusions

In summary, this study preliminarily suggests that features derived from hand and eye movement data analysis in the virtual kiosk test hold potential as digital markers for early screening of patients with MCI. An SVM trained on 4 key behavioral features in the virtual kiosk test, namely hand movement speed, proportion of fixation duration, time to completion, and the number of errors, was able to differentiate between patients with MCI and healthy controls with statistical significance. The model achieved high accuracy (93.3%), sensitivity (100%), specificity (83.3%), precision (90%), *F*_1_-score (94.7%), and AUC (0.98). These key behavioral features were also found to be closely associated with various cognitive domains through correlation analysis. Our findings suggest that digital markers, specifically the analysis of hand and eye movements in VR, have the potential of screening patients with MCI from an embodied cognition perspective. This is why we call for further research to confirm the reliability and validity of this approach.

## Appendix

Multimedia Appendix 1 Demonstration video of the virtual kiosk test performed by a real patient.

Multimedia Appendix 2 Performance of the classifiers using hand movement speed, proportion of fixation duration, time to completion, and the number of errors.

## Abbreviations

AD: Alzheimer disease
AUC: area under the receiver operating characteristic curve
DST: F+B: Digit Span Test: Forward+Backward
IADL: instrumental activities of daily living
K-TMT-E: B: Korean-Train Making Test-Elderly’s version Part B
MCI: mild cognitive impairment
MRI: magnetic resonance imaging
RCFT: Rey Complex Figure Test
S-K-BNT: Short form of the Korean-Boston Naming Test
SNSB-C: Seoul Neuropsychological Screening Battery-Core
SVLT-E: DR: Seoul Verbal Learning Test-Elderly’s version: Delayed Recall
SVM: support vector machine
VR: virtual reality
