# Supplementary material for: Digital Marker for Early Screening of Mild Cognitive Impairment Through Hand and Eye Movement Analysis in Virtual Reality Using Machine Learning: First Validation Study
Source: J Med Internet Res. 2023 Oct 20;25:e48093. doi: 10.2196/48093 (PMC10625097; doi:10.2196/48093)
Supplement: Multimedia Appendix 2 [file jmir_v25i1e48093_app2.docx]

**Table S1**. Performance of the classifiers using hand movement speed, proportion of fixation duration, time to completion, and the number of errors.

| Classifier model | Accuracy (%) | Sensitivity (%) | Specificity (%) | Precision (%) | *F*_1_-score (%) |
| --- | --- | --- | --- | --- | --- |
| SVM^a^ | 93.3 | 100 | 83.3 | 90 | 94.7 |
| XGBoost^b^ | 86.7 | 88.9 | 83.3 | 88.9 | 88.9 |
| Decision Tree | 80 | 77.8 | 83.3 | 87.5 | 82.4 |
| Random Forest | 73.3 | 77.8 | 66.7 | 77.8 | 77.8 |

^a^SVM: support vector machine. This model was selected as the best model.
^b^XGBoost: extreme gradient boosting.
